# Interaction between human placental microvascular endothelial cells and a model of human trophoblasts: effects on growth cycle and angiogenic profile

**DOI:** 10.1002/phy2.244

**Published:** 2014-03-26

**Authors:** Weston Troja, Kicheol Kil, Charles Klanke, Helen N. Jones

**Affiliations:** 1 Division of Pediatric Surgery, Cincinnati Children's Hospital Medical Center, Cincinnati, Ohio; 2 Department of Obstetrics and Gynecology, The Catholic University of Korea, Seoul, South Korea; 3 Division of Reproductive Sciences, Cincinnati Children's Hospital Medical Center, Cincinnati, Ohio

**Keywords:** Angiogenesis, co‐culture, human placenta microvascular endothelial cells, IGF‐1, Trophoblast

## Abstract

Intrauterine growth restriction (IUGR) is a leading cause of perinatal complications, and is commonly associated with reduced placental vasculature. Recent studies demonstrated over‐expression of IGF‐1 in IUGR animal models maintains placental vasculature. However, the cellular environment of the placental chorionic villous is unknown. The close proximity of trophoblasts and microvascular endothelial cells in vivo alludes to autocrine/paracrine regulation following Ad‐HuIGF‐1 treatment. We investigated the co‐culturing of BeWo Choriocarcinoma and Human Placental Microvascular Endothelial Cells (HPMVECs) on the endothelial angiogenic profile and the effect Ad‐HuIGF‐1 treatment of one cell has on the other. HPMVECs were isolated from human term placentas and cultured in EGM‐2 at 37°C with 5% CO_2_. BeWo cells were maintained in Ham's F12 nutrient mix with 10% FBS and 1% pen/strep. Co‐cultured HPMVECS+BeWo cells were incubated in serum‐free control media, Ad‐HuIGF‐1, or Ad‐LacZ at MOI 0 and MOI 100:1 for 48 h. Non‐treated cells and mono‐cultured cells were compared to co‐cultured cells. Angiogenic gene expression and proliferative and apoptotic protein expression were analysed by RT‐qPCR and immunocytochemistry, respectively. Statistical analyses was performed using student's *t*‐test with *P *<**0.05 considered significant. Direct Ad‐HuIGF‐1 treatment increased HPMVEC proliferation (*n *=**4) and reduced apoptosis (*n *=**3). Co‐culturing HPMVECs+BeWo cells significantly altered RNA expression of the angiogenic profile compared to mono‐cultured HPMVECs (*n *=**8). Direct Ad‐HuIGF‐1 treatment significantly increased Ang‐1 (*n *=**4) in BeWo cells. Ad‐HuIGF‐1 treatment of HPMVECs did not alter the RNA expression of angiogenic factors. Trophoblastic factors may play a key role in placental vascular development and IGF‐1 may have an important role in HPMVEC growth.

## Abstract (precis)

Cellular environment of the placental chorionic villous is relatively unknown. Co‐culture of human placental microvascular endothelial cells and model trophoblasts demonstrated two things: the overexpression of HuIGF‐1 in the microvascular endothelial cells alters the cell growth cycle, and trophoblastic factors alter the angiogenic profile of the microvascular endothelial cells.

## Introduction

In the human placenta following terminal villous differentiation, multiple cell types exist in close proximity (Gude et al. 2004) and may undergo paracrine regulation following an insult or treatment of another cell type. Classic in vitro studies involve the culture of one cell type such as placental trophoblast; while useful, these types of studies may not reflect the in vivo environment per se. Previous studies have used conditioned medium to model paracrine signaling between multiple placental cell types associated with establishment of placentation and invasion on the maternal side, however the models do not demonstrate the co‐culture of two cell types in close proximity in vivo, nor do any represent the villous tree of the fetal side of the placenta (Zhou et al. 2003).

Placental microvascular cells form the fetal capillaries of the villous tree, whereas syncytiotrophoblasts are exposed to maternal blood and both of these may interact with mesenchymal stromal cells or cytotrophoblasts within the placenta milieu (Charnock‐Jones and Burton 2000; Gude et al. 2004). Due to close proximity, sometimes even sharing a basal membrane in late gestation (Gude et al. 2004), these two cell types may influence each other in response to signals from either the fetal, maternal or both circulations.

Proper development of the placenta depends upon autocrine and paracrine signaling between trophoblasts and vascular endothelial cells (Charnock‐Jones and Burton 2000; Ong et al. 2000). Studies of vasculogenic and angiogenic signaling such as the VEGF‐A/VEGFR‐2 and Ang‐1/Tie2 ligand/receptor mechanisms demonstrate the close interaction between the cells (Ahmed and Perkins 2000; Charnock‐Jones and Burton 2000; Dunk et al. 2000) and suggest potential cross‐talk between the cells may be necessary for appropriate growth, development, and remodeling. Advancement in understanding the cellular environment of the placental chorionic villous has been delayed by the lack of an in vitro model that generates appropriate cell interaction (Forbes et al. 2008).

Intrauterine growth restriction (IUGR) is one of the major causes of perinatal morbidity and mortality, affecting 7~15% of all pregnancies (Cetin and Alvino 2009). One structural alteration commonly associated with IUGR is the reduced fetal vasculature of the placenta (Ahmed and Perkins 2000). In IUGR pregnancies, reductions in fetal growth are associated with reduced fetal cord serum levels of IGF‐1 (Nieto‐Díaz et al. 1996; Klauwer et al. 2011), which reflect compensation for the altered substrate (Halhali et al. 2000) and oxygen supply (Dinleyici et al. 2006). Similarly, potential therapies administered via the maternal circulation such as IGF‐1 in guinea pig models of IUGR also impact placental growth and function and may alter the fetal microvasculature as well as the trophoblast (Sferruzzi‐Perri et al. 2007).

Work in our laboratory demonstrated that over‐expression of human Insulin‐like Growth Factor‐1 (HuIGF‐1) following intra‐placental injection of adenoviral human IGF‐1 (Ad‐HuIGF‐1) in the placenta corrects fetal weight deficits in animal models of IUGR (Keswani et al. 2004; Chung et al. September 2010) and increases placental cross‐sectional area (Katz et al. 2009). Intra‐placental Ad‐HuIGF‐1 injection increased both vascular area and labyrinth volume in the mouse model maintaining area and volume at sham levels. Furthermore, recent studies demonstrated over‐expression of HuIGF‐1 in BeWo Choriocarcinoma cells stimulates proliferation and reduces apoptosis (Jones et al. 2013a). Currently, it is unknown whether the noted increase in vascular area results from a direct effect of Ad‐HuIGF‐1 on placental endothelial cells or an indirect effect of adjacent trophoblastic cells when treated with Ad‐HuIGF‐1 (Chung et al. September 2010). This study investigated the effects of co‐culturing BeWo cells (a model of human trophoblasts) and human placenta microvascular endothelial cells (HPMVECs) on angiogenic profile and if treatment of one cell type by adenoviral‐mediated HuIGF‐1 impacted cellular responses of the other.

## Material and Methods

### Isolation of HPMVECs

Placentas from normal term pregnancies after caesarean section were collected (*n *=**6) under IRB approval (Good Samaritan Hospital and Cincinnati Children's Hospital Medical Center). Placenta was manually dissected and freed from the amniotic sac and maternal decidua under aseptic conditions. Small fragments (Sakurai et al. 2012) were cut out and washed twice in Hanks' balanced salt solution (HBSS). The minced tissues were digested for 2 h in a shaking incubator at 37°C in 0.28% (W/V) of collagenase II, 0.25% dispase II and 2.5 mg DNase (type I) in Hepes‐buffered HBSS (Dunk et al. 2012). After incubation, the cell suspension was collected and filtered using 100 *μ*m mesh. Filtrate was centrifuged at 600×*g* for 7 min. RBC lysis buffer was added to the cell pellet for 10 min at room temperature. The reaction was stopped with PBS, cells were centrifuged 5 min at 300×*g* and the supernatant discarded and washed with PBS. Washed tissue was filtered through a 40 *μ*m pore size Nylon mesh. The cells were centrifuged for 5 min at 300×*g*, resuspended with 1 mL of EBM‐2 Basal medium (Lonza, Allendale, NJ), and counted. CD31‐coated Dynabeads (Invitrogen) were used to isolate endothelial cells. The bead‐bound cells were plated on dishes coated with attachment factor (AF) (Thermo Fisher, Waltham, MA) and cultured in EGM‐2 (Lonza, Allendale, NJ). Experiments were conducted on cells at passages 3–8.

### HPMVECs characterization

Isolated HPMVECs were tested for their expression of endothelial cell markers CD31 and von Willebrand factor, and for smooth muscle cell contamination using immunocytochemistry.

### Human placental microvascular endothelial cell culture

Human placental microvascular endothelial cells were maintained at 37°C and 5% CO_2_ in EGM‐2. Cell culture flasks were treated with AF prior to use. Cells were sub‐cultured every 7 days and medium was replaced every 2 days.

### BeWo cell culture

BeWo Choriocarcinoma cells were routinely maintained in nutrient Mixture F‐12 Hams medium (Sigma, St. Louis, MO), penicillin (100 U/mL), streptomycin (100 *μ*g/mL), and 10% fetal bovine serum at 37°C under 5% CO_2_. Cells were sub‐cultured every 7 days and the medium was replaced every 2 days. Experiments were conducted on cells at passages 5–15.

### Viral vectors

Ad‐HuIGF‐1 and Ad‐LacZ constructs were obtained from Dr. M. Herlyn (Institute for Human Gene Therapy, University of Pennsylvania). Both constructs are replication defective, serotype 5 adenovirus vectors (Keswani et al. 2004). Ad‐LacZ and Ad‐HuIGF‐1 were given at an MOI of 100:1 for 24 h in serum‐free media. Control cells were incubated in serum‐free media (MOI 0) for the same timeframe.

### Ad‐HuIGF‐1 treatment

Direct treatment in co‐culture was considered the treatment of one cell type with adenovirus at MOI 100 in serum free media. In contrast, indirect treatment in co‐culture was considered the effects the directly treated cells had on the untreated cells in co‐culture.

#### HPMVEC mono‐culture

Human placenta microvascular endothelial cells were seeded onto AF‐ treated 60 mm 6‐well plates or 4‐chamber culture slides in EGM‐2. After 24 h, medium was changed to EBM‐2 Basal medium and treated directly with either control EBM‐2 at MOI 0, Ad‐LacZ or Ad‐HuIGF‐1 at an MOI 100:1. After a further 48 h, media and cell lysate were collected for analysis.

#### Direct HPMVEC and BeWo co‐culture model

Human placenta microvascular endothelial cells were plated onto AF‐treated 4‐chamber culture slides in EGM‐2. After 16 h, BeWo cells that had been pre‐treated with control EBM‐2, Ad‐HuIGF‐1, or Ad‐LacZ for 24 h, were added to the chambers of the culture slide in EBM‐2. Cells were co‐cultured for a further 48 h, and effects on proliferation and apoptosis of both cell types were assessed by immunocytochemistry. The procedure was reversed for untreated BeWos with pre‐treated HPMVECs except BeWos were initially cultured in complete serum F‐12 Ham's medium then switched to EBM‐2.

#### Indirect HPMVEC and BeWo co‐culture model

**Figure 1.**
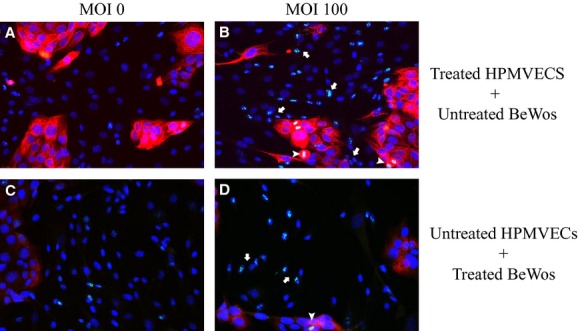
Direct co‐culture of BeWo cells and HPMVECs. Ki67 (green) stained with anti‐Ki67. Nuclei stained with DAPI (blue). BeWo cells (red) stained with cytokeratin‐7. HPMVECs represented by blue nuclei without red matrix. (A and C) Control BeWo and HPMVECs demonstrating staining and that the cell lines can survive together in co‐culture. (B) Ki67 expression increased in HPMVECs (white arrows) and BeWo cells (white arrowheads) when only HPMVECs treated with Ad‐HuIGF‐1. (D) Ki67 expression increased in HPMVECs and BeWo cells when only BeWo treated with Ad‐HuIGF‐1.

Cell culture inserts of 0.4 *μ*m pore size 30 mm diameter (Millipore, Bedford, MA) were placed in a 60 mm 6‐well plate, treated with AF and seeded with 2.5 × 10^5^ BeWo cells/insert in complete Nutrient Mixture F‐12 Ham medium. Concurrently, a fresh 6‐well plate was AF treated and seeded with 2.5 × 10^5^ HPMVECs/well in complete EGM‐2. 24 h later the BeWo and HPMVEC media was changed to EBM‐2 and the BeWo cells were treated with control EBM‐2 at MOI 0, Ad‐LacZ, or Ad‐HuIGF‐1 at an MOI 100:1. After 24 h, media was removed from BeWo and HPMVEC cultures, BeWo cell inserts were moved onto respective HPMVEC wells, and new EBM‐2 was added into the wells and inserts as depicted in Figure 1. Following a further 48 h incubation, media and cell lysates were collected for analysis. The procedure was reversed for untreated BeWos and treated HPMVECs, however the cells remained on the same surfaces.

### Immunocytochemistry

All antibodies used were obtained from, Abcam Inc., Cambridge, MA unless otherwise noted. Cells were fixed in 1% paraformaldehyde for 20 min. After washing, non‐specific binding was blocked by incubation with 5% normal goat serum. To confirm cell phenotype, the cells were incubated with anti‐von Willebrand factor (1:1000), anti‐CD‐31 (1:100) and anti‐Actin B4 (1:500) (Seven Hills Bioreagents, Cincinnati, OH) antibodies. For analysis of proliferation and apoptosis, cells were incubated with anti‐Ki‐67 (1:100) and anti‐caspase‐3 (1:750), respectively. A rabbit normal immunoglobulin fraction or primary antibody omittance (Abcam) served as control. After incubation, direct co‐culture slides were washed and incubated with anti‐mouse cytokeratin‐7 antibody (1:750) for 2 h at room temperature to aid cell identification. After extensive washing, the slides were incubated with appropriate secondary antibodies, washed again, mounted with Prolong Gold with DAPI (Invitrogen) and examined using fluorescence or bright‐field microscope (Nikon, Melville, NY).

### RNA isolation

Cells were lysed using RLT Buffer from Qiagen Inc. (Valencia, CA). RNA was isolated using the RNeasy Mini Kit, QIAshredder, and on‐column DNA digest (Qiagen) following manufacturer's protocol. RNA quantification and quality control was assessed using Nanodrop Spectrophotometer (Thermo Fisher).

### Reverse transcription quantitative polymerase chain reaction

Expression levels of angiogenic genes in HPMVECs for all experimental conditions were determined quantitatively by real‐time RT‐qPCR. 1 *μ*g of purified RNA was reverse‐transcribed into cDNA using Hi‐Cap cDNA Conversion Reaction on MJ Research PTC 200 Thermal Cycler (MJ Research Inc., St. Bruno, Canada) following manufacturer's protocol.

Primers for human TBP, ACTB, VEGF‐A, PlGF, Ang‐1, and Ang‐2 were designed using Primer‐BLAST (NCBI.com) for specificity (Table 1). Gene expression was assayed with a melt curve, in duplicate, using 1/40th of the cDNA template and 300 nmol/L of forward and reverse primer in a 25 µL Power SYBR Green PCR Master Mix reaction in the Applied Biosystems StepOne‐Plus Real‐Time PCR System. cDNA in each well was normalized to reference genes Human *β*‐Actin (Hu ACTB) and Human TATA Binding Protein (Hu TBP). Relative quantification expression levels were calculated by comparative C_T_ method (Pfaffl) via StepOne Software v2.3 and normalized to smallest value of control single cell culture.

**Table 1.** Angiogenic profile and housekeeping primer sequences used in RT‐qPCR.

Oligonucleotides	Sequences (5′‐3′)	Amplicon (bp)	Intron (bp)
Hu TBP F	GAACCACGGCACTGATTTTC	77	2285
Hu TBP R	TGCCAGTCTGGACTGTTCTTC		
Hu ACTB F	CGCGAGAAGATGACCCAG	75	441
Hu ACTB R	TAGCACAGCCTGGATAGCAA		
Hu Ang‐1 1F	TGATGGACACAGTCCACAAC	79	18,528
Hu Ang‐1 1R	TCTTCCTCTCTTTTTCCTCCCT		
Hu Ang‐2 1F	GATTTTGGACCAGACCAGTGA	84	6144
Hu Ang‐2 1R	TTGTCTTCCATAGCTAGCACC		
Hu VEGF‐A 2F	GAGGGCAGAATCATCACGAA	72	3076
Hu VEGF‐A 2R	TCTCGATTGGATGGCAGTAG		
Hu PlGF 1F	GAGGAGAGAGAAGCAGAGA	120	3479
Hu PlGF 1R	GTGACGGTAATAAATACACGAG		
Hu‐spec IGF1 3F	TTTTGTGATTTCTTGAAGGTGA	104	4519
Hu‐spec IGF1 3R	CGGTCCAGCCGTGGCAGA		

### Statistical analysis and data presentation

Data presented as mean ± SD. Statistical analysis was performed according to student's *t*‐test (Excel, Microsoft). Level of significance (*P*‐value) was defined as 0.05 or less.

## Results

Daily observations demonstrated HPMVECs and BeWo cells were viable and in good condition when cultured in EBM‐2 for 3–5 days and together in co‐culture. Immunocytochemistry shown in Figure 1A–D demonstrates our observations.

### Proliferation of directly and indirectly Ad‐HuIGF‐1 treated cells

**Figure 2.**
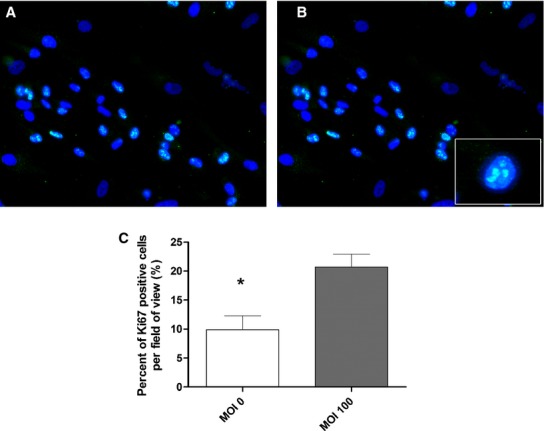
Ki67 expression (green) and localization in HPMVEC mono‐culture. (A) MOI 0 control demonstrating some Ki67 expression. (B) Ki67 expression is increased around HPMVECs following Ad‐HuIGF‐1 treatment at MOI 100. (C) Percent of Ki67 expression is significantly increased per field of view following exposure of HPMVECs to Ad‐HuIGF‐1 100:1. Student's *t*‐test, *P <**0.05, mean ± SD.

**Figure 3.**
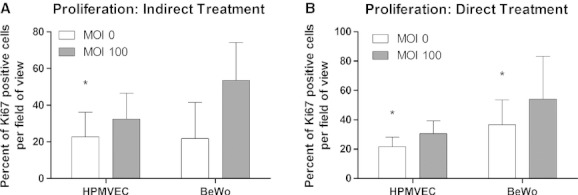
Ki67 expression of HPMVECs and BeWo cells in indirect co‐culture. (A) Percent of Ki67 expression in HPMVECs and BeWo cells increases significantly following Ad‐HuIGF‐1 treatment of other cell type. (B) Percent of Ki67 expression increases significantly in HPMVECs and BeWo cells following Ad‐HuIGF‐1 treatment on same cell type. Student's *t*‐test, *P <**0.05, mean ± SD.

Based on the percent of Ki67 expressed per field of view, direct Ad‐HuIGF‐1 treatment on HPMVECs significantly increased the proliferation of HPMVECs in mono‐culture (*P *=**0.028, *n *=**4, Fig. 2B and C) compared to control untreated HPMVECs (Fig. 2A and C) suggesting IGF‐1 may effect HPMVEC growth cycle. A trend of increased proliferation in HPMVECs was seen in co‐culture in indirectly treated HPMVECs (*n *=**4); however this increase did not reach significance (Fig. 3A). Directly Ad‐HuIGF‐1 treated HPMVECs increased proliferation in co‐culture with untreated BeWo cells compared to untreated co‐culture (*P *=**0.045, *n *=**3, Fig. 3B).

Both indirect (*P *=**0.006, *n *=**3, Fig. 3A) and direct (*P *=**0.024, *n *=**3, Fig. 3B) treatments of co‐cultured BeWos cells significantly increased proliferation compared to control indicative of some form of paracrine signalling between cell types.

### Apoptosis of directly and indirectly Ad‐HuIGF‐1 treated cells

**Figure 4.**
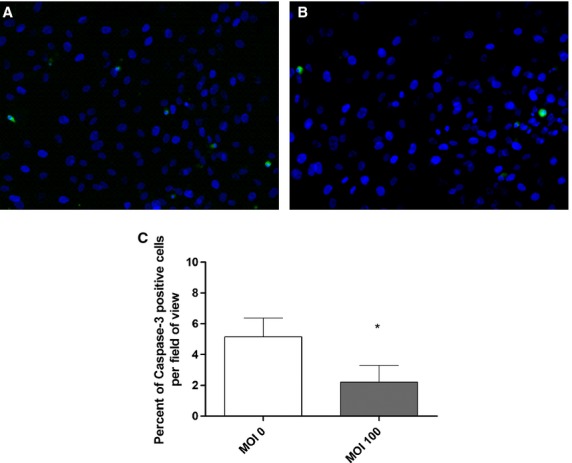
Cas‐3 expression (green) and localization in HPMVEC mono‐culture. (A) MOI 0 control demonstrating some Cas‐3 expression. (B) Ki67 expression is decreased around HPMVECs following Ad‐HuIGF‐1 treatment at MOI 100. (C) Percent of Cas‐3 expression is significantly decreased per field of view following exposure of HPMVECs to Ad‐HuIGF‐1. Student's *t*‐test, *P <**0.05, mean ± SD.

**Figure 5.**
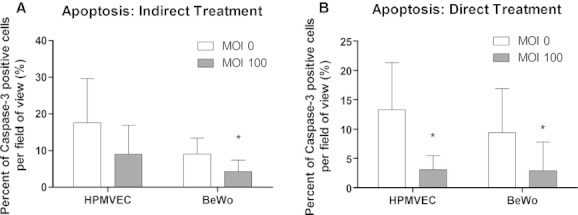
Cas‐3 expression of HPMVECs and BeWo cells in indirect co‐culture. (A) Percent of Cas‐3 expression in HPMVECs and BeWo cells reduces significantly in BeWo cells following indirect Ad‐HuIGF‐1 treatment. (B) Percent of Cas‐3 expression reduces significantly in HPMVECs and BeWo cells following direct Ad‐HuIGF‐1 treatment. Student's *t*‐test, *P <**0.05, mean ± SD.

Direct Ad‐HuIGF‐1 treatment of mono‐cultured HPMVECs significantly reduced apoptosis of HPMVECs (*P *=**0.035, *n *=**3, Fig. 4A–C) according to Cas3 expression of immunocytochemistry. Similarly, apoptosis of HPMVECs was reduced after directly Ad‐HuIGF‐1 treated HPMVECs were co‐cultured with non‐treated BeWo cells compared to untreated co‐culture (*P *=**0.036, *n *=**3, Fig. 5B). When HPMVECs were indirectly treated by directly treated BeWo cells, apoptosis in HPMVECs was not significantly reduced (*n *=**4); however there was a strong trend for reduced apoptosis (Fig. 5A).

Both direct (*P *=**0.019, *n *=**3, Fig. 5B) and indirect (*P *=**0.019, *n *=**3, Fig. 5A) treatments of co‐cultured BeWo cells significantly reduced in apoptosis based on the percent of Caspase‐3 per field of view compared to control co‐culture.

### Angiogenic gene expression in HPMVEC is altered when co‐cultured with BeWo cells

**Figure 6.**
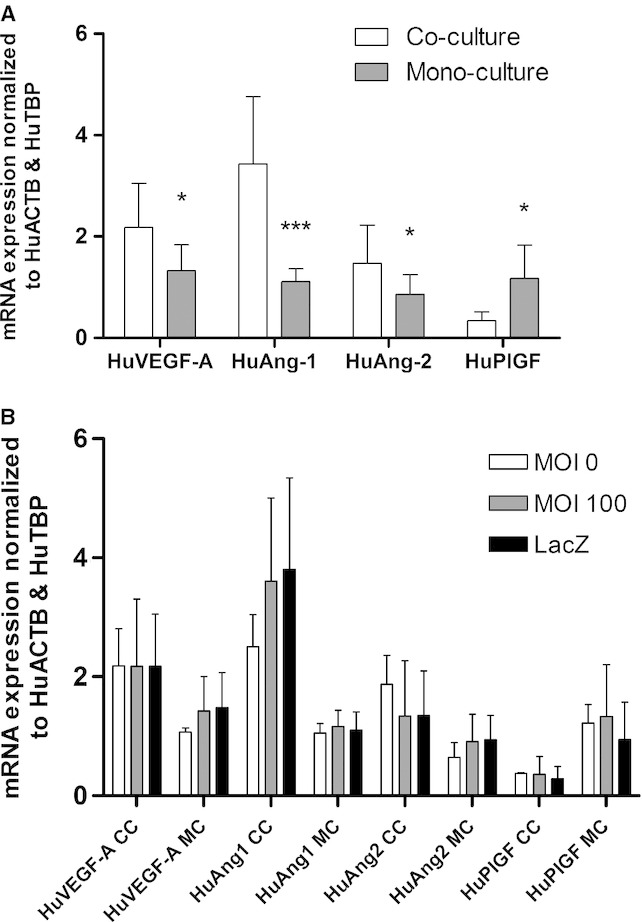
Summary of mRNA expression of angiogenic profile in HPMVECs from indirect co‐culture. (A) Indirectly co‐cultured HPMVECs compared to mono‐cultured HPMVECs. (B) Difference in mRNA expression of co‐cultures (CC) and mono‐cultures (MC) treated with MOI 0 (control), Ad‐HuIGF‐1 at MOI 100, or Ad‐LacZ at MOI 100. Student's *t*‐test, *P <**0.05, ***P <**0.005, mean ± SD.

Using RT‐qPCR, we analyzed gene expression of angiogenic growth factors angiopoietin‐1 (Ang‐1), angiopoietin‐2 (Ang‐2), vascular endothelial growth factor‐A (VEGF‐A), and placental growth factor (PlGF) in both the mono‐cultures and the indirect co‐culture model. Direct co‐culture was not used for this experiment since extracting RNA from a single cell type was not feasible. In comparison to the mono‐culture, indirectly co‐culturing HMPVEC and BeWo cells significantly increased expression levels of Ang‐1 (*P *=**0.0001, *n *=**8), Ang‐2 (*P *=**0.037, *n *=**8), and VEGF‐A (*P *=**0.019, *n *=**8), and significantly reduced PlGF expression (*P *=**0.025, *n *=**5) in the HPMVECs (Fig. 6A).

### Ad‐HuIGF‐1 effect on angiogenic gene expressions of HPMVECs

No viral effect was seen on the angiogenic profiles of HPMVECs as demonstrated by no change in gene expression following exposure to Ad‐LacZ. Furthermore, direct treatment of the HPMVECs with Ad‐HuIGF‐1 did not result in significant changes in angiogenic profile expression. Similarly, indirect treatment of HPMVECs with Ad‐HuIGF‐1 caused no significant differences in VEGF‐A, Ang‐1, Ang‐2, or PlGF mRNA expression (Fig. 6B).

### Ad‐HuIGF‐1 significantly increased angiopoietin‐1 in BeWo cells

**Figure 7.**
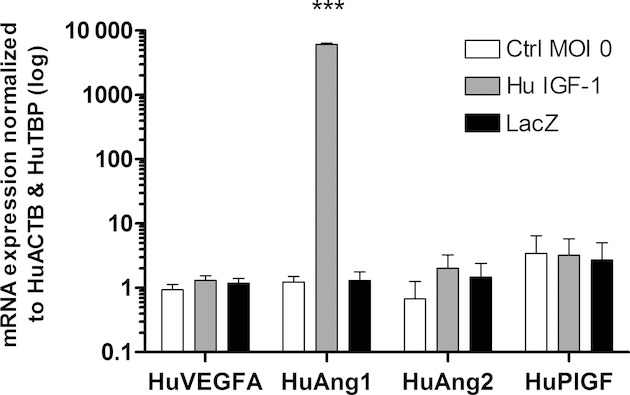
mRNA expression of angiogenic factors in BeWo cells directly treated with Ad‐HuIGF‐1 at MOI 100. ANOVA, Post hoc Tukey's test, ***P <**0.005, mean ± SD.

Trophoblast angiogenic mRNA expression was analyzed using RT‐qPCR. Compared to control, direct Ad‐HuIGF‐1 treatment of BeWo cell significantly increased Ang1 expression in BeWo cells (*P *=**0.000000007, *n *=**4, Fig. 7). However, there was no change in the expression levels of the other angiogenic genes analyzed following Ad‐HuIGF‐1 exposure.

## Discussion

The present study establishes both an in vitro co‐culture model representative of the terminal villous of the human placental villous tree and the regulation of HPMVECs following co‐culture or Ad‐HuIGF‐1 treatment. Co‐culture of HPMVECs and BeWo cells altered the angiogenic factor profile of HPMVECs by increasing VEGF‐A, Ang‐1, and Ang‐2 and reducing PlGF gene expression suggesting cross‐talk between the cells may regulate angiogenesis in HPMVECs at the transcriptional level. Both direct and indirect Ad‐HuIGF‐1 treatments on HPMVECs and BeWo cells led to increased proliferation and reduced apoptosis, supporting the hypothesis that HuIGF‐1 may have an autocrine and paracrine role in cellular processes. The Ad‐HuIGF‐1 treatment of BeWo cells upregulated Ang‐1 gene expression; whereas treatment of HPMVECs with Ad‐HuIGF‐1 had no effect on angiogenic gene expression in HPMVECs. The results support the concept that trophoblastic factors regulate microvascular endothelial cells.

This is the first study to successfully co‐culture HPMVECs with a model of human trophoblasts and the first co‐culture characteristic of the terminal villous. Many previous studies have co‐cultured placenta derived cells (Lash et al. 2011; Sakurai et al. 2012; Tien et al. 2012), even recapitulating the epithelial‐stromal organization in the chorionic villous (Forbes et al. 2008); however none investigated a co‐culture using HPMVECs or depicting the terminal villous. By directly co‐culturing the HPMVECs and BeWo cells together, we provide proof that the cells are compatible together and the cellular environment is suitable.

The increased proliferative response combined with the reduced apoptotic response observed in directly and indirectly Ad‐HuIGF‐1 treated HPMVECs demonstrate IGF‐1 is biologically active within the cultures. We have previously exhibited this in BeWo cells directly treated with Ad‐HuIGF‐1 that showed increased proliferation and invasion, reduced apoptosis, and altered functionality of glucose and amino acid transporters (Jones et al. 2013a)–(2013b). Similarly, in this study we have shown direct Ad‐HuIGF‐1 treatment can also increase proliferation and reduce apoptosis in HPMVECs. This response in HPMVECs indicates that IGF‐1 influences the growth cycle of the cell, which likely corresponds with Miller et al. (2005), Forbes et al. (2002), and Jones et al. (2013b) who previously demonstrated IGF‐1 regulates the growth cycle and function of other placental cell lines.

In co‐culture, the proliferative and apoptotic data demonstrates the effect the Ad‐hIGF‐1 treatment of one cell types had on the other. The responses in indirectly treated HPMVECs were not significant; however there was a strong trend indicative of cell growth. The increased proliferation and reduced apoptosis of the indirectly treated cells indicates a form of paracrine response is likely occurring between the HPMVECs and BeWo cells. The data is encouraging because it suggests the treatment of either HPMVECs or trophoblasts can impact the cellular responses of the other. The IGF‐1 mechanism in the placenta and HPMVECs is unknown, but autocrine and paracrine processes may derive from the phosphorylation of insulin‐like growth factor‐1 receptor (Holmes et al. 1999; Imrie et al. 2012) and activation of the MAPK and PI3K pathways as was shown in other microvascular endothelium (Johansson et al. 2008) and placenta cells (Forbes and Westwood 2008; Forbes et al. 2008).

Furthering our findings from the proliferation assays, we evaluated the angiogenic gene response of HPMVECs to Ad‐HuIGF‐1 and the presence of model trophoblasts. Upregulation of VEGF‐A, Ang‐1, and Ang‐2 and downregulation of PlGF in co‐cultured HPMVECs suggests cellular cross‐talk may regulate angiogenesis at the level of transcription. Transcriptional regulation is consistent with the findings of Geva et al. (2002). The differences in gene expression of VEGF‐A, Ang‐1, and Ang‐2 may be due to secreted factors from the trophoblast cells. One factor may be PlGF, which is highly expressed in trophoblasts (Shore et al. 1997; Arroyo et al. 2004). This growth factor has pro‐angiogenic roles in both cell types and functions in paracrine fashion (Yang et al. 2003) by binding to VEGFR‐1 (flt‐1) as a homodimer or as a PlGF/VEGF heterodimer (Shore et al. 1997; De Falco 2012). Cell specific PlGF‐mediated signal transduction was found in trophoblast and endothelial cells (Arroyo et al. 2004); and given the abundant expression level of PlGF in placenta, more specifically in trophoblasts, HPMVECs may have PlGF specific signal transductions too. Our results suggest exogenous PlGF may stimulate angiogenic gene expression and negatively regulate endogenous PlGF expression in HPMVECs. The increase of VEGF‐A is probably the cause of the altered angiogenic profile since angiopoietins are regulated downstream of VEGF‐A (Ahmed and Perkins 2000; Singh and Milner 2009; Singh et al. 2011). The cause of the increase is unclear but could come from exogenous PlGF activation of the HIF‐1*α* pathway or perhaps VEGF‐A and endogenous PlGF working inversely.

Studies have shown the angiopoietin‐Tie signaling pathway in endothelial cells is tightly regulated by VEGF‐A at the protein ectodomain (Findley et al. 2007; Singh et al. 2011). Binding of Ang‐1 to Tie2 stimulates the PI3K/Akt pathway and Akt activation results in Ang‐1 and Ang‐2 expression (Thurston and Daly 2012). In relation to our study, we speculate alterations in this pathway may explain why a response to the exogenous Ang‐1 from the Ad‐HuIGF‐1 treated BeWo cells is not seen. If protein only increased in one of the cells, the origin of Ang‐1 would be clear, however since both cells increased in mRNA, which cell the activating mRNA derives from is uncertain. Nonetheless, the secretion of Ang‐1 and PlGF by trophoblasts indicates trophoblasts may take on a perivascular cell‐like role to the HPMVECs, and therefore the interaction between these two cells requires further investigation.

Interestingly, Ad‐HuIGF‐1 treatment of HPMVECs demonstrates IGF‐1 was not associated with an increase in angiogenic gene expression, which is surprising given the observations that IGF‐1 enhances the angiogenic response in human endothelial cells (Nicosia et al. 1994; Roesel and Nanney 1995; Su et al. 2003). Our results are in agreement with the proliferative and apoptotic responses stimulated by IGF‐1 documented in these previous publications, but differ with the angiogenic responses. These studies base their findings of IGF‐1 promoting angiogenesis in endothelial cells on structural observations rather than the alteration of angiogenic gene expression. Angiogenesis is not a means of proliferation, but rather the formation of blood vessels, therefore the lack of modulation of angiogenic gene expression by IGF‐1 indicates IGF‐1 may regulate angiogenesis in a post‐transcriptional manner.

In summary, trophoblastic factors transcriptionally regulate HPMVEC angiogenic gene expression. The underlying autocrine and paracrine mechanisms between the two cells in the placental terminal villous are unknown and require further investigation. Congruently, the autocrine and paracrine mechanisms of IGF‐1 on HPMVEC may enhance cell growth and could have a defining role in placental vascularization.

## Acknowledgments

We would like to thank Dr. M. Herlyn, University of Pennsylvania, Philadelphia, PA for supplying the Adenoviral vectors.

## Conflict of Interest

There is no conflict of interest by the authors.
